# A cross-sectional observational study to assess socio-demographic factors in newly diagnosed TB DM comorbidity

**DOI:** 10.12688/f1000research.122471.3

**Published:** 2022-08-18

**Authors:** Rashmi Hullalli, M R Gudadinni, Rohith Motappa

**Affiliations:** 1 Department of Community Medicine, Shri B M Patil Medical College, Vijayapura, Karnataka, 586103, India; 2 Department of Community Medicine, Kasturba Medical College Hospital, Mangalore, Manipal Academy of Higher Education, Manipal, India

**Keywords:** Diabetes mellitus, prevalence, risk factors, tuberculosis

## Abstract

**Background:** Tuberculosis (TB) and diabetes mellitus (DM) co-morbidity is one of the rising public health problems. There is growing evidence that DM is an important risk factor for TB. This study was carried out to know the prevalence of DM among newly detected sputum positive pulmonary TB patients registered in District Tuberculosis Centre and to assess the risk factors of DM among TB patients.

**Methods:** In a cross-sectional study, newly detected sputum positive pulmonary TB patients were screened for DM (those having symptoms of DM). Furthermore, they were diagnosed by detecting blood glucose levels (≥200 mg/dL). Mean, standard deviation (SD), Chi-squared and Fisher-Freeman-Halton exact tests were used to determine the significant associations. P-values less than 0.05 were considered to be statistically significant.

**Results:** A total of 215 TB patients were included in this study. The prevalence of DM among TB patients was found to be 23.7% (2.8% known and 97.8% new cases). Significant associations were found between age (>46 years old), educational status, smoking habits, alcohol consumption, physical activity, presence of DM symptoms and family history of DM.

**Conclusions:** Routine screening for DM is mandatory due to its increasing prevalence, which may help in early diagnosis and to reduce complications by proper management that in turn helps in the successful outcome of TB treatment.

## Introduction

Diabetes mellitus (DM) has become more common as a result of urbanisation, as well as social and economic development. People who have a weakened immune system, such as those who have diabetes, are more likely to proceed from latent to active disease. ^1^ When compared to individuals without DM, people with DM have a two to three times higher risk of tuberculosis (TB). ^2^ DM is associated with about 10% of all TB cases worldwide. ^1^ A high percentage of individuals living with DM and TB go undiagnosed or are diagnosed too late. As a result, early discovery can aid in improving the care and control of both disorders. ^3^

If a four-drug intensive phase regimen is changed to a two-drug regimen after two months in the presence of culture-positive time, DM can lengthen the time it takes for sputum culture conversion, resulting in the development of drug resistance. ^4^ People with DM and TB have a higher risk of death during therapy and of TB relapse once treatment is completed. The presence of infectious illnesses, such as TB, complicates DM. ^5^ Glycaemic management has also been shown to improve treatment outcomes in TB patients. According to recent studies, DM accounts for 20% of smear-positive TB cases, ^2^ and that an increase in DM prevalence in India has posed a significant barrier to TB reduction. ^6^

There is a lack of literature regarding this comorbidity in Vijayapura Taluk. Therefore, this study was conducted to determine the percentage of DM among TB patients in Vijayapura Taluk, to study the socio-demographic profile of TB patients with DM and to identify potential risk factors.

## Methods

### Ethical approval

Ethical approval was obtained from the Institutional Ethical Committee of Shri B M Patil Medical College, BLDE University (Certificate No – 65/21-10-17; dated 21.09.2017). All participants provided written informed consent, which was obtained as part of the questionnaire during data collection. Informed consent forms ^7^ and information sheets ^8^ can be found as *Extended data.*

### Study design and setting

The present study was a cross-sectional study performed at Vijayapura Tuberculosis Unit, Karnataka. It covers 14 primary health centres, four urban health centres and two medical colleges. A TB register was used to approach the TB patients. Sputum-positive TB patients in Vijayapura taluk registered from January 1 ^st^ to December 31 ^st^, 2016, over the age of 18 years old were included. The study was carried out between March 2017 and February 2018. Patients who were critically ill, not willing to participate, or women who were pregnant or lactating were not included in the study. Considering the prevalence of DM among TB patients is estimated to be 30.6%, ^3^ at 95% confidence level and at 20% allowable error, the sample size was calculated by using the following formula:

$$n=\frac{Z^{2}\times p\times q}{d^{2}}=215$$ (1)

Non-diabetic patients were screened for DM by examining blood glucose levels in capillary blood using a finger-prick glucometer. Those found to be positive and having symptoms of DM were further evaluated by determining blood glucose levels. Health workers/Accredited Social Health Activist (ASHAs) were involved in the study, and objectives were explained to them. The TB patients were approached at their homes/Directly observed treatment, short-course (DOTS) centres/Primary Health Centres (PHCs) with the help of the Senior Treatment Supervisor. The study purpose was explained to participants at the time of the questionnaire. They were informed that their participation in the study was voluntary and that they could withdraw from the study at any point. Maintenance of confidentiality about data and findings was assured to the participants and their consent was obtained. Participants were tracked using ID numbers to maintain anonymity. Data were collected on proforma and only the investigator had access to it.

### Study tools

A semi-structured, pretested questionnaire was developed and administered to newly detected sputum-positive pulmonary TB patients, with modifications relevant to local conditions. ^9^ The pilot study was performed on 30 individuals for pretesting. Statistical validation for the questionnaire was done by Cronbach’s Alpha.

The procedure included the following four parts: i) Socio-demographic variables; ii) anthropometric measurements; iii) blood glucose estimation and iv) evaluation of any risk factors leading to the occurrence of DM.

Instruments that were be used for general physical examination: i) A measuring tape; ii) weighing machine and iii) stethoscope. All these instruments were regularly standardized throughout the period of data collection.

### Variables

*Measurement of height, weight and body mass index (BMI)*

Height was taken using a measuring tape in centimetres (cm) and recorded to the nearest 0.5 cm. Weight was measured in kilograms (kg) using a standardized bathroom weighing machine and was recorded to the nearest 0.5 kg. In this study, BMI classification proposed by the World Health Organization (WHO) Western Pacific Regional Office in collaboration with the International Obesity HTask Force (IOTF) steering committee (2000) ^10^ for Asian people was used. It is also known as the Quetelet Index and was used to assess obesity.

*Diagnosis of DM*

All participants, including those who were not diabetic but had symptoms of DM (polydipsia, polyuria, weight loss) were checked for random blood glucose levels. Classification of DM was done using the American Diabetic Association criteria. ^11^

Criteria for the diagnosis of DM included symptoms of DM plus random blood glucose concentration ≥11.1 mmol (200 mg/dL) OR fasting plasma glucose ≥7 mmol (126 mg/dL) OR Haemoglobin A1c ≥6.5% OR two-hour plasma glucose ≥11.1 mmol (200 mg/dL) during an oral glucose tolerance test.

### Data analysis

The data were compiled in a Microsoft Excel 2010 (RRID:SCR_016137) work sheet and analysed using SPSS version 16.0 software (RRID:SCR_002865). The data were presented in the form of tables and graphs wherever necessary. All characteristics were summarized descriptively. For continuous variables, the summary statistics of number, mean, and standard deviation (SD) about the arithmetic mean were used. For categorical data, the number and percentage were used in the data summarized. Chi-squared test was used to know the significant associations. P-values less than 0.05 were considered to be statistically significant. Univariate regression analysis was used to detail the risk factors in the development of DM.

## Results

Table 1 ^12^ shows that out of 215 study participants, the majority (27%) of them belonged to the age group of 26–35 years old followed by 46–55 (21.9%) and 36–45 (20.9%) years old. In our study, men constituted 64.2% (138) of participants and women constituted 35.8% (77). A total of 88% of the TB patients were from rural backgrounds. Furthermore, 89.8% of the study participants were married, while only 7.9% were unmarried and there were about 2.3% widowed participants in the study. A total of 87.9% of study participants belonged to Hindu religion and the rest of them *i.e.*, 12.1% belonged to the Muslim community. More than half (54.4%) of the study participants studied up to primary school followed by secondary school (23.7%), while 14.9% were illiterate in the study. In this study, 87% (187) of the participants belonged to a nuclear family, while 13% (28) were from a joint family. In our study, 48.8% of the participants belonged to the lower class according to modified BG Prasad classification, ^13^ followed by 25.6% in the middle class and 20% in the lower middle class.

**Table 1.** Distribution of socio-demographic characteristics of study participants (n=215).

Study variables	Number (n)	Percentage (%)
**Age, years**		
15–25	32	14.9
26–35	58	27.0
36–45	45	20.9
46–55	47	21.9
56–65	26	12.0
>65	7	3.3
**Sex**		
Male	138	64.2
Female	77	35.8
**Place**		
Rural	190	88.0
Urban	25	12.0
**Marital status**		
Married	193	89.8
Unmarried	17	7.9
Widowed	5	2.3
**Religion**		
Hindu	189	87.9
Muslim	26	12.1
**Education**		
Illiterate	32	14.9
Primary	117	54.4
Secondary	51	23.7
Pre-University College (PUC)	14	6.5
Graduate	1	0.5
**Type of family**		
Nuclear	187	87.0
Joint	28	13.0
**Socioeconomic class**		
Upper class	4	1.9
Upper middle class	8	3.7
Middle class	55	25.6
Lower middle class	43	20.0
Lower class	105	48.8

Out of 215 study participants, 74% (159) were in the continuation phase (CP) of the TB treatment and 26% (56) were in the intensive phase (IP).

Table 2 details the prevalence of anti-TB treatment adherence. It was found that nearly 10.6% of the study participants were non-adherent to the treatment course.

**Table 2.** Distribution of cases according to treatment adherence (n=215).

Treatment adherence	Number (n)	Percentage (%)
No	23	10.6
Yes	192	89.4
Total	215	100.0

**Figure 1.**
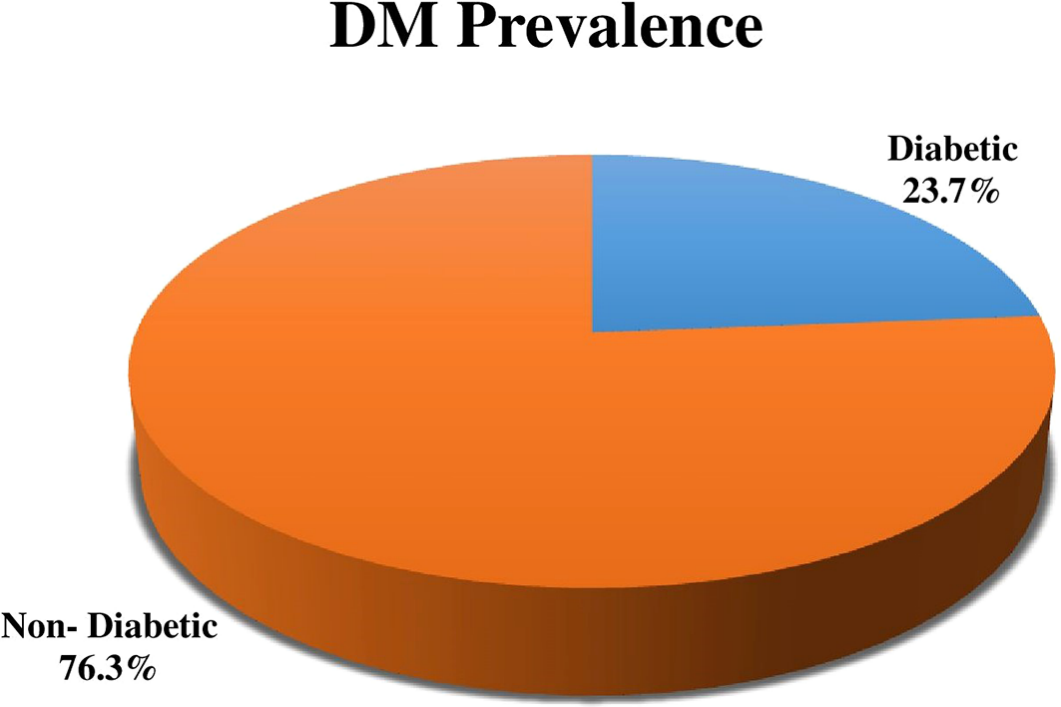
Distribution of the prevalence of diabetes mellitus (DM) among tuberculosis (TB) patients.

Out of 215 study participants the percentage of DM was 23.7% (51) ( Figure 1).

When looking at the food habits of the study participants, 49% were vegetarians and 51% used to consume both vegetarian and non-vegetarian food items. A total of 43.1% of the study participants were current smokers and 51% were ex-smokers, while 5.9% were non-smokers. A majority (70.6%) of the study participants did not consume alcohol and about 29.4% consumed alcohol. Most (90.2%) of the study participants were engaged in moderate physical activity, while 3.9% described themselves as sedentary and 5.9% engaged in heavy physical activity ( Table 3).

**Table 3.** Distribution of behavioural patterns among participants with diabetes mellitus (DM) (n=51).

Study variables	Number (n)	Percentage (%)
**Food habits**		
Vegetarian	25	49
Mixed	26	51
**Smoking**		
Current smoker	22	43.1
Ex-smoker	26	51
Non-smoker	3	5.9
**Alcohol consumption**		
Consumed alcohol	15	29.4
Did not consume alcohol	36	70.6
**Occupation**		
Non-government	39	76.5
Government	4	7.8
Housewife	7	13.7
Student	1	2
**Physical activity**		
Sedentary	2	3.9
Moderate	46	90.2
Heavy	3	5.9

Univariate regression analysis was applied to ascertain the percentage of DM and its associated risk factors ( Table 4). It was found that participants in the age group of 56–65 years old, who were educated until the 7 ^th^ standard, who were ex-smokers, those who consumed alcohol and who lead a sedentary lifestyle were the highest risk factors associated with the occurrence of DM, when compared to their counter variables. All these values were statistically significant.

**Table 4.** Univariate regression analysis detailing the risk factors in the development of diabetes mellitus (DM).

Demographic information	Diabetic		Non-diabetic		Odd’s ratio	95% CI	p-value
	n	%	n	%			
**Age, years**							0.001
15-25	1	2.0	31	18.9	0.037	0.0046–0.2913	
26-35	3	5.9	55	33.5	0.062	0.0169–0.2265	
36-45	7	13.7	38	23.2	0.2093	0.0078–0.5629	
46-55®	22	43.1	25	15.2	®		
56-65	15	29.4	11	6.7	1.550	0.5895–4.073	
>65	3	5.9	4	2.4	0.8523	0.1725–4.235	
**Educational status**							0.01
Illiterate	11	21.6	21	12.8	8.368	2.117–33.183	
7th std	34	66.7	83	50.6	6.554	1.910–22.493	
10th std ®	3	5.9	48	29.3	®		
12th std	3	5.9	11	6.7	4.634	0.7739–24.605	
Graduate	0	0.0	1	0.6	4.619	0.1572–135.7	
**Smoking habits**							0.049
Current smoker	2	3.9	8	4.9	1.034	0.2084–5.134	
Ex-smoker	20	39.2	36	22.0	2.299	1.164–4.451	
Non smoker®	29	56.9	120	73.2	®		
**Alcohol habits**							0.002
Consumed alcohol	15	29.4	19	11.6	3.180	1.473–6.683	
Did not consume alcohol	36	70.6	145	88.4	®		
**Physical activity**							0.007
Sedentary	2	3.9	5	3.0	21.383	0.2596–7.365	
Moderate®	46	90.2	159	97.0	®		
Heavy	3	5.9	0	0.0	4.011	1.217–473.61	

® = reference value, std = standard.

## Discussion

Our study showed that majority of the TB patients were in the age group of 26-35 years old (27%). Similar results were found in a study done by Damtew *et al.*, ^14^ in Addis Ababa Ethiopia where the majority of patients were 25–44 years of age. A study done by Balakrishnan *et al.*, ^15^ in Kerala revealed that most of the patients were 45–54 years of age. Other studies done by Kishan *et al.*, ^16^ in Patiala Punjab and Dutt *et al.*, ^17^ in Ahemdabad reported that the 40–60 years old age group was the most commonly involved. These differences in age groups may be due to the different location of the study and study design setting. The mean age was found to be 52.1 years old among diabetics and 37.1 years old among non-diabetics, which was similar to a study done by Natarajaboopathy *et al.*, ^18^ in Tamil Nadu where the mean age of the DM TB patients was 52.92 years old and was statistically significant. Padmalatha *et al.*, ^3^ in Andhra Pradesh showed that the mean age was 46.5±10.3 years old among diabetics and 35.8±11.7 years old among non-diabetics.

More than half (54.4%; 117) of the TB patients studied up to primary schooling, 23.7% (51) of patients completed high school education and 14.9% (32) of patients were illiterate, where it was observed that most of the TB patients had received less schooling, which was consistent with other studies. A study done by Sarker *et al*., ^19^ in Bangladesh reported that 25.1% of the participants had primary schooling, 19.8% had secondary schooling and 40.7% were illiterate. Another study by Tahir *et al*., ^20^ in Pakistan showed that 51.6% of participants were illiterate, 34.7% had primary schooling and 10.7% had secondary schooling.

In our study, 4.7% of participants were current smokers and 69.3% were non-smokers, which was in line with a study done by Damtew E *et al*., ^14^ in Ethiopia, which revealed that 15% of subjects were smokers and 85% were non-smokers. However, another study done by Ekeke *et al.*, ^21^ in Nigeria showed that 4.8% of participants were non-smokers and 95.2% were current smokers. This variation may be due to different social scenarios.

We found that the majority (84.2%) of the TB patients did not consume alcohol and about 15.8% used to consume alcohol, which was similar to a study by Viswanathan *et al.*, ^22^ in Tamil Nadu in which 38.9% did not consume alcohol and 1.6% consumed alcohol. While another study by Damtew E *et al.*, ^14^ in Ethiopia determined that there wasn’t much difference in alcohol consumption *i.e.*, 51.7% did not consume alcohol and 48.3% consumed alcohol, which may be due to different socio-cultural factors.

The percentage of DM among TB patients in our study was 23.7%, which is consistent with the reports of other studies ^23^ done in Karnataka State in 2011 where the prevalence was 32%, in Kerala State, 44% (2012) in Tamil Nadu State, and 25% in India (2012). Other studies like an institutional based cross-sectional study done by Padmalatha *et al*., ^3^ in Andhra Pradesh showed the prevalence of DM as 30.6%. A facility based cross-sectional study done by Raghuraman *et al.*, ^24^ in Puducherry (2017) reported the DM prevalence to be 29%. In contrast to the aforementioned findings, another study done in Nigeria by Olayinka *et al.*, ^25^ found the prevalence to be 5.7%, which could be attributed to differences in demographic characteristics. We employed the American Diabetic Association (ADA) criteria to assess the percentage of DM and studied TB patients registered under the Revised National TB Control Programme (RNTCP).

Some of the variables analysed were based on the information obtained by the study participants hence an element of recall bias and masking of data could be present. TB patients registered under RNTCP were included in the study, thus patients being treated in private hospitals may have missed. Among all the TB patients, only new sputum positive pulmonary TB patients were included, which may be a limitation. In the present study, only a few risk factors of DM were studied. Other risk factors could not be studied due to a lack of resources. The percentage of DM among TB patients was 23.7%, which is quite high so screening of all TB patients should be done just like HIV screening in order to aid in early diagnosis and proper management of the disease. For DMTB patients, regular blood glucose estimation and treatment should be given in DOTS centres along with anti-TB drugs. Primordial prevention can play an important role in preventing the occurrence of DM. Health education regarding the risk factors and symptoms of DM should be given to high-risk groups.

Although the findings were found to be consistent with previously reported studies, direct comparisons are not valid for the reasons stated above, as well as the fact that researchers used different criteria to diagnose the conditions over time, the non-representativeness of patients studied in terms of number and selection criteria, and the different settings of the research. A well-designed large-scale observational study or meta-analysis could resolve the problem.

## Conclusions

Routine screening for DM in TB patients should be mandatory due to increases in the prevalence of DM, which would not only help in early diagnosis, but also reduces complications by proper management and in turn will help in the successful outcome of TB treatment.

### Limitations of the study

1. Due to time constraints and logistic problems we couldn’t test all the tuberculosis patients. It will be incorporated into our further research.
2. Only a selected sub population of symptomatic patients were tested.
3. Mean Blood Glucose values were not recorded and it is one of the limitations of our study.

## Data availability

### Underlying data

Figshare: Percentage of Diabetes Mellitus among newly detected sputum positive Pulmonary Tuberculosis patients and associated risk factors. https://doi.org/10.6084/m9.figshare.19878115.v1. ^12^

### Extended data

Figshare: Questionnaire.docx. https://doi.org/10.6084/m9.figshare.20014160.v1. ^9^

Figshare: Consent form.docx. https://doi.org/10.6084/m9.figshare.20014172.v1. ^7^

Figshare: Information sheet for the study participant.docx. https://doi.org/10.6084/m9.figshare.20014175.v1. ^8^

Data are available under the terms of the Creative Commons Attribution 4.0 International license (CC-BY 4.0).
