# Supplementary material for: A cross-sectional observational study to assess socio-demographic factors in newly diagnosed TB DM comorbidity
Source: F1000Res. 2024 May 20;11:674. Originally published 2022 Jun 20. [Version 4] doi: 10.12688/f1000research.122471.4 (PMC11109703; doi:10.12688/f1000research.122471.4)
Supplement: Peer review history

# Reviewer report: Reviewer response for version 3

**DOI:** 10.5256/f1000research.137402.r195369

**Published:** 2024-05-07

**Authors:** Rajesh Mondal

**Affiliations:** 1 ICMR - Bhopal Memorial Hospital & Research Centre, Bhopal, India

The percentage of DM among TB patients is 23.7%. It is consistent with the earlier reports published in this area. How come the rise of DM in India (in recent years) will not affect the prevalence/percentage of TB?

 Did you find any other comorbidity like HIV? 

 Any other non-communicable or communicable diseases?

Is the work clearly and accurately presented and does it cite the current literature?

Yes

If applicable, is the statistical analysis and its interpretation appropriate?

Yes

Are all the source data underlying the results available to ensure full reproducibility?

Yes

Is the study design appropriate and is the work technically sound?

Yes

Are the conclusions drawn adequately supported by the results?

Yes

Are sufficient details of methods and analysis provided to allow replication by others?

Yes

Reviewer Expertise:

TB

I confirm that I have read this submission and believe that I have an appropriate level of expertise to confirm that it is of an acceptable scientific standard.

# Reviewer report: Reviewer response for version 2

**DOI:** 10.5256/f1000research.137253.r147253

**Published:** 2022-08-11

**Authors:** Anil Kapur

**Affiliations:** 1 World Diabetes Foundation, Bagsvaerd, Denmark

The authors have changed the title, added limitations and removed a table but as stated in my review, the continued use of the term 'prevalence' in the context of the study is incorrect. That term has been used extensively all through the paper to describe their findings. Based on the methods employed the study should not be using the term 'prevalence' as it is misleading. At best the authors can state that in our study sample XX% of TB patients had DM. 

 I have nothing further to add.

Is the work clearly and accurately presented and does it cite the current literature?

Partly

If applicable, is the statistical analysis and its interpretation appropriate?

Partly

Are all the source data underlying the results available to ensure full reproducibility?

Partly

Is the study design appropriate and is the work technically sound?

Partly

Are the conclusions drawn adequately supported by the results?

Partly

Are sufficient details of methods and analysis provided to allow replication by others?

Partly

Reviewer Expertise:

Diabetes, and Diabetes and TB co-morbidity

I confirm that I have read this submission and believe that I have an appropriate level of expertise to confirm that it is of an acceptable scientific standard, however I have significant reservations, as outlined above.

# Response

**Published:** 2022-08-11

**Authors:** Rohith Motappa

**Affiliations:** Kasturba Medical College Hospital, India

Respected Sir,

 As suggested by you, the phrase "Prevalence " is replaced with "Percentage", wherever it is applicable.

 Thanks

# Reviewer report: Reviewer response for version 1

**DOI:** 10.5256/f1000research.134460.r141524

**Published:** 2022-07-07

**Authors:** Navya Nagendra

**Affiliations:** 1 Department of Community Medicine, Yenepoya Medical College, Mangaluru, Karnataka, India

The study is a relevant study as DM (diabetes mellitus) plays a role in treatment process of TB. However there are few comments which can be addressed in the manuscript.

1. In study tools the use of stethoscope for this study is not justified.
2. It was mentioned non DM patients were first screened by using glucometer and further evaluated by determining blood glucose levels. However, it is not clear which parameters was considered for definitive diagnosis? In the diagnosis section it is mentioned FBS (fasting blood sugar) or PPBS (post prandial blood sugar) or HbA1C. Which test/tests for confirmation did the investigator ask for when RBS (random blood sugar) tested positive using glucometer?
3. In results it is not mentioned how many new cases of diabetes mellitus was diagnosed by the investigators during the study process. 
4. Discussion can be elaborate stating the possible reasons for the results obtained. 
5. Conclusion can be written better as presently it looks more like recommendations. 
6. Kindly check for incorrect spelling like Ahmedabad in discussion. 

Is the work clearly and accurately presented and does it cite the current literature?

Yes

If applicable, is the statistical analysis and its interpretation appropriate?

Yes

Are all the source data underlying the results available to ensure full reproducibility?

Partly

Is the study design appropriate and is the work technically sound?

Yes

Are the conclusions drawn adequately supported by the results?

Partly

Are sufficient details of methods and analysis provided to allow replication by others?

Partly

Reviewer Expertise:

TB, Tobacco control, Primary health care

I confirm that I have read this submission and believe that I have an appropriate level of expertise to confirm that it is of an acceptable scientific standard.

# Reviewer report: Reviewer response for version 1

**DOI:** 10.5256/f1000research.134460.r141525

**Published:** 2022-06-30

**Authors:** Anil Kapur

**Affiliations:** 1 World Diabetes Foundation, Bagsvaerd, Denmark

The authors have made an attempt to study the prevalence of DM among patients being treated for TB as well as understand the socio demographic factors.

1. While the authors indicate a prevalence of 23.7%, this may not be accurate as they tested only so called symptomatic cases. It is well known that type 2 diabetes very rarely presents with the classical symptoms of polyuria, polydipsia etc. Moreover, symptoms such as weight loss, tiredness and fatigue, change in appetite etc. are similar to what is seen in TB and therefore ascribed to TB. Therefore estimating prevalence based on testing a subsample of symptomatic population is likely to underestimate the problem.
2. The authors state that DM comorbidity was higher in older population, however in the discussion they talk about age of the whole population and compare it with studies which are talking about age of patients with DM co-morbidity, so it is difficult to understand how their study is different?
3. The authors could have enhanced their study analysis by describing what was the diabetes diagnosis rate in people in the intensive treatment and continuous treatment phase. It is known that hyperglycemia may reduce even without antihyperglycemic treatment as the acute phase of TB comes under control and weight loss caused by TB helps improve glycemic control especially in those who were overweight. This again points towards the methodical flaw in testing only symptomatic cases. 
4. The authors state " *DM is associated with about 10% of all TB cases worldwide.*" without providing a credible reference. " *DM accounts for 20% of smear-positive TB cases*" but no reference is given to support this. 
5. Overall the article does not add any further insight on the topic of DM-TB comorbidity except describing the prevalence of DM among TB patients in Vijayapura TB Unit in Karnataka, even this may not be accurate given that only a selected sub population of symptomatic patients were tested. 
6. There is no data on mean blood glucose values of those diagnosed with DM and those without DM. 

Based on the review and the methodological problems the article cannot be justifiably titled as a prevalence of DM among newly diagnosed... at best it can be titled "a cross sectional observational study to assess socio demographic factors in newly diagnosed TB DM comorbidity".

Is the work clearly and accurately presented and does it cite the current literature?

Partly

If applicable, is the statistical analysis and its interpretation appropriate?

Partly

Are all the source data underlying the results available to ensure full reproducibility?

Partly

Is the study design appropriate and is the work technically sound?

Partly

Are the conclusions drawn adequately supported by the results?

Partly

Are sufficient details of methods and analysis provided to allow replication by others?

Partly

Reviewer Expertise:

Diabetes, and Diabetes and TB co-morbidity

I confirm that I have read this submission and believe that I have an appropriate level of expertise to confirm that it is of an acceptable scientific standard, however I have significant reservations, as outlined above.

# Response

**Published:** 2022-07-17

**Authors:** Rohith Motappa

**Affiliations:** Kasturba Medical College Hospital, India

Respected Anil Kapur Sir,

 Thank you very much for the review of our manuscript. We sincerely appreciate all the valuable comments and suggestions given by you, which helped us to improve the quality of the article. Our responses to the Reviewers’ comments are described below in a point-to-point manner

1. Due to time constraints and logistic problems we couldn’t test all the tuberculosis patients. It will be incorporated into our further research.
2. Noted sir, in our study, we have more prevalence among the aged population, and that comparison will be removed and others will be kept.
3. We couldn’t check the diagnostic rate in the intensive or continuation phase, hence that image will be removed, however, it will be given prime importance in our next study.
4.  References are as follows:
  1. Revised National TB Control Programme Technical and Operational Guidelines for Tuberculosis Control in India 2016.India. p 269.
    ​​​​​​​
  2. Park K. Epidemiology of Communicable Diseases. In: Textbook of Preventive and Social Medicine. 24th edition. Jabalpur, India: M/s Banarsidas Bhanot Publishers; 2017.p.185-216.
5. That is accurate sir, we will modify accordingly.
  ​​​​​​​
6. Mean Blood Glucose values were not recorded and it is one of the limitations of our study.

Thanks and Regards
